# Regulation through condensation: sRFR1 condensates in the upper lateral root cap control root growth

**DOI:** 10.1093/plcell/koag001

**Published:** 2026-01-07

**Authors:** Gwendolyn K Kirschner

**Affiliations:** Assistant Features Editor, The Plant Cell, American Society of Plant Biologists, United States; The James Hutton Institute, Invergowrie, Dundee DD2 5DA, United Kingdom

For years, biologists have known that the localization of a protein influences its behavior, but a new type of cellular compartment has complicated the story. Biomolecular condensates act as enigmatic pockets where proteins can be trapped, protected, or activated. Within biocondensates, proteins and their ligands can be sequestered from their target, thereby inhibiting a reaction in the cell; or vice versa, the sequestration can increase the dwell time and enhance interactions and reactions (Field et al. 2023).

In new work, **Jianbin Su and colleagues (Su et al. 2025)** show that, beyond its established role as a negative regulator of effector-triggered immunity, the SUPPRESSOR of rps4-RLD1 (SRFR1) functions within biomolecular condensates in the Arabidopsis root meristem. Besides enhanced resistance to bacteria and stunted shoot phenotypes (Bhattacharjee et al. 2011), *srfr1* mutants also had shorter roots, raising the question of how SRFR1 regulates root growth.

Longitudinal root growth is governed by cell division and elongation in the root meristem, located at the root tip. In this region, small undifferentiated cells divide frequently until they leave the meristem shootward, where they stop dividing and start to elongate, facilitating root growth (Fig. 1) (Dolan et al. 1993). This transition zone is established by a balance of the phytohormones auxin and cytokinin. Auxin transport and biosynthesis are regulated by ethylene, influencing cell elongation (Vaseva et al. 2018). In *srfr1* mutants, shorter roots were correlated with a shorter root meristem, dependent on ethylene signaling. Surprisingly, while SRFR1 was localized in the cytoplasm in the epidermis, it localized to puncta in upper lateral root cap (LRC) cells, suggesting condensate formation. The LRC acts as an auxin sink, and programmed cell death releases auxin to initiate lateral roots shootward of the transition zone (Di Mambro et al. 2019). Aligned with that, SRFR1 only formed condensates in living cells of the upper LRC adjacent to cells that had undergone programmed cell death (Fig. 1), suggesting a function related to transition zone formation.

**Figure 1:**
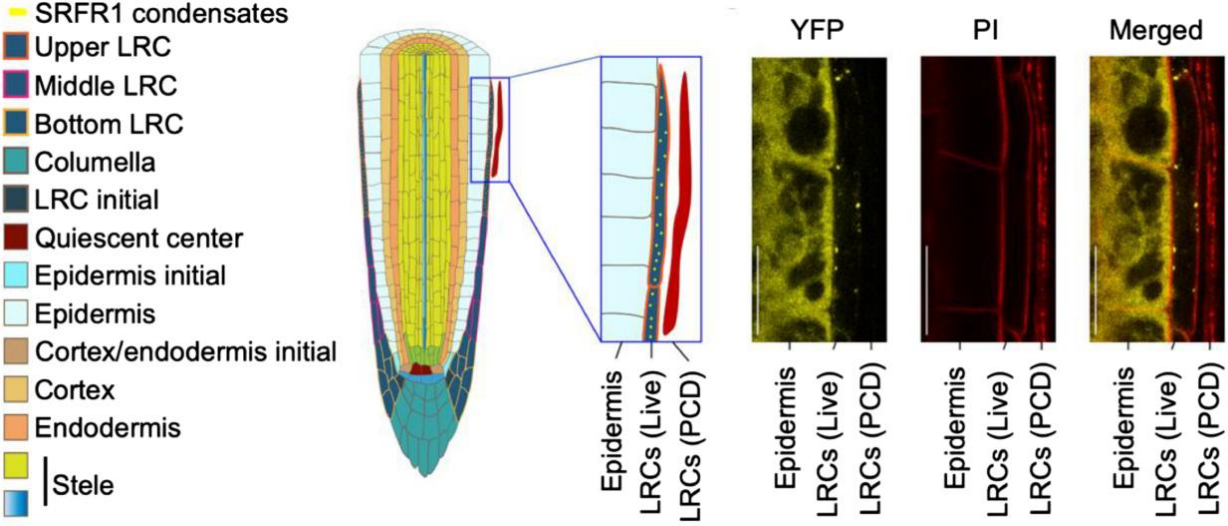
SRFR1 forms condensates in the upper LRC cells. The upper LRC cells cover the transition zone of the root meristem outside the epidermis (left). In epidermis cells, SRFR1-YFP is localized in the cytoplasm, but in the upper LRC cells adjacent to LRC cells that have undergone programmed cell death (PCD) it forms condensates (right). Adapted from Su et al. (2025), Figure 3.

The growth of *srfr1* roots was inhibited more when grown in solid medium or in liquid culture compared to being exposed to air, correlated with a decrease in condensate accumulation. Treatment with ethylene precursors, auxin, and cytokinin also decreased condensate accumulation, while gibberellic acid treatment and blocking ethylene production promoted it, suggesting regulation by environmental and hormonal cues.

Expression of SRFR1 proteins with different deleted domains showed that the plant-associated N-terminal tetratricopeptide repeat (PANT) domain, along with its adjacent intrinsically disordered region IDR1, functioned as a condensation module. IDRs do not adopt a stable ordered secondary structure and are often associated with condensate formation (Field et al. 2023). In SRFR1, IDR1 prevented PANT domain aggregation at high temperatures while promoting PANT polymerization at low temperatures. This chaperone-like function was facilitated by its zwitterionic nature, that is, containing an equal number of positively and negatively charged functional groups. Adding positive charges to IDR1 enhanced SRFR1 condensate formation and further promoted root growth.

Immunoprecipitation-mass spectrometry analysis identified 63 proteins enriched in the SRFR1 condensates, including several proteins associated with plasma membrane functions. Based on their findings, the authors propose that live upper LRCs containing SRFR1 condensates function as a buffering barrier, protecting epidermal cells in the transition zone from auxin oscillations caused by programmed cell death of the outer LRC. This buffering capacity may be achieved, for example, by modulating the activity of proteins sequestered within the condensates. One such candidate is a purine uptake permease, which is likely involved in regulating cytokinin uptake into LRC cells.

The findings indicate that the upper LRC–specific, thermally stable yet stimuli-responsive SRFR1 condensates play a key role in promoting optimal root growth. This function might be particularly important during early spring, when daily temperature fluctuations are pronounced.

**Recent related articles in**  ***The Plant Cell***:

- Ruiz-Solaní et al. (2023) analyzed how the metacaspase MC1 is recruited into biomolecular condensates (stress granules) upon proteotoxic stress in Arabidopsis.
- Field et al. (2023) found that the chloroplast-localized RNA-binding protein CP29A undergoes phase separation in a temperature-dependent manner mediated by its prion-like domain.
- Safi et al. (2023) developed a system for the visualization of protein–protein interactions and kinase activities in planta based on phase separation.
- Legen et al. (2024) reviewed current research on biomolecular condensates in plant development and discuss challenges and opportunities for further research.

## Dive Curated Terms

The following phenotypic, genotypic, and functional terms are of significance to the work described in this paper:

SRFR1 Gramene: AT4G37460
SRFR1 Araport: AT4G37460
